# Engineering Metabolism of Chimeric Antigen Receptor (CAR) Cells for Developing Efficient Immunotherapies

**DOI:** 10.3390/cancers13051123

**Published:** 2021-03-05

**Authors:** Joslyn L. Mangal, Jamie L. Handlos, Arezoo Esrafili, Sahil Inamdar, Sidnee Mcmillian, Mamta Wankhede, Riccardo Gottardi, Abhinav P. Acharya

**Affiliations:** 1 Biological Design Graduate Program, School for Biological and Health Systems Engineering, Arizona State University, Tempe, AZ 85281, USA; jmangal@asu.edu; 2 Department of Chemical Engineering, School for the Engineering of Matter, Transport, and Energy, Arizona State University, Tempe, AZ 85281, USA; jhandlos@asu.edu (J.L.H.); aesrafil@asu.edu (A.E.); sahil.inamdar@asu.edu (S.I.); smcmill4@asu.edu (S.M.); mamta.wankhede@asu.edu (M.W.); 3 Department of Pediatrics, Division of Pulmonary Medicine, Perelman School of Medicine, University of Pennsylvania, Philadelphia, PA 19104, USA; GOTTARDIR@email.chop.edu; 4 Fondazione Ri.MED, 90133 Palermo, Italy; 5 Department of Materials Science and Engineering, School for the Engineering of Matter, Transport, and Energy, Arizona State University, Tempe, AZ 85281, USA; 6 Biodesign Center for Immunotherapy, Vaccines and Virotherapy, Tempe, AZ 85281, USA

**Keywords:** CAR macrophage, CAR T cell, immunotherapy, solid tumors, immunometabolism, tumor microenvironment

## Abstract

**Simple Summary:**

This review paper here describes the recent progress that has been made in chimeric antigen receptor (CAR) -based therapies for treatment of tumors and the role of metabolism in the tumor microenvironment in relation to these therapies. Moreover, this manuscript also discusses role of different CAR-based cells for treatment of solid tumors, which is a major challenge in the CAR immunotherapy field.

**Abstract:**

Chimeric antigen receptor (CAR) T cell-based therapies have shown tremendous advancement in clinical and pre-clinical studies for the treatment of hematological malignancies, such as the refractory of pre-B cell acute lymphoblastic leukemia (B-ALL), chronic lymphocytic leukemia (CLL), and large B cell lymphoma (LBCL). However, CAR T cell therapy for solid tumors has not been successful clinically. Although, some research efforts, such as combining CARs with immune checkpoint inhibitor-based therapy, have been used to expand the application of CAR T cells for the treatment of solid tumors. Importantly, further understanding of the coordination of nutrient and energy supplies needed for CAR T cell expansion and function, especially in the tumor microenvironment (TME), is greatly needed. In addition to CAR T cells, there is great interest in utilizing other types of CAR immune cells, such as CAR NK and CAR macrophages that can infiltrate solid tumors. However, the metabolic competition in the TME between cancer cells and immune cells remains a challenge. Bioengineering technologies, such as metabolic engineering, can make a substantial contribution when developing CAR cells to have an ability to overcome nutrient-paucity in the solid TME. This review introduces technologies that have been used to generate metabolically fit CAR-immune cells as a treatment for hematological malignancies and solid tumors, and briefly discusses the challenges to treat solid tumors with CAR-immune cells.

## 1. Introduction

**Figure 1:**
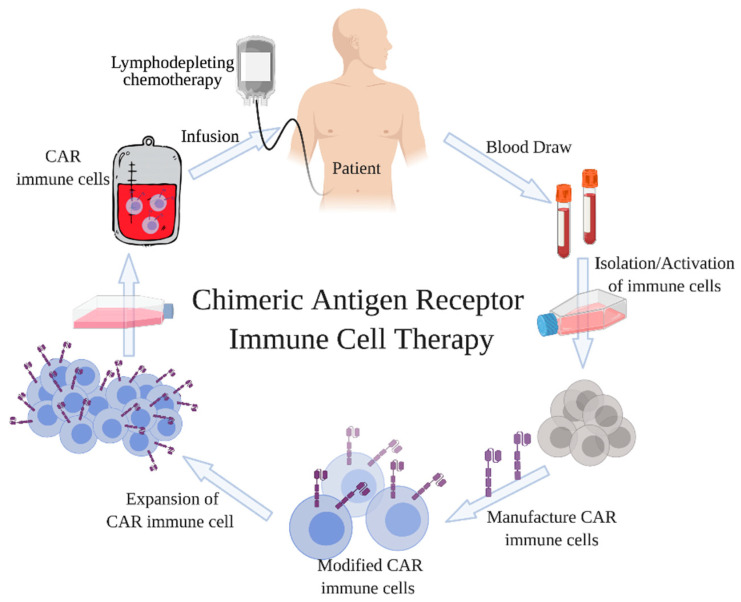
Treatment of patients with CAR immune cells. In the first step, immune cells are isolated from the patient’s blood by leukapheresis. These autologous immune cells are then manufactured off-site and genetically modified to target and kill antigen carrying cancer cells. The treatment is initiated by intravenous infusion of CAR immune cells into the patient.

Adoptive cell transfer (ACT) strategies including tumor-infiltration lymphocytes (TILs), T cell receptor (TCR) engineered T cells, and CAR T cells have been highly efficacious cancer immunotherapies in clinic. CAR T cells are a type of cell-based therapy where autologous T lymphocytes are genetically engineered to express the binding site of specific antibodies for the ability to target tumor-associated antigens (TAAs) [1]. There have been three generations of CAR T cells to date. In the first generation of CARs, the T-cell signaling domain was fused with an intracellular portion of a TCR CD3ζ subunit [2]. However, their poor performance in vivo, due to lack of co-stimulation, led to the development of second-generation CAR T cells where two types of T-cell signaling domains, a co-stimulatory domain, either CD28 or 4-1BB, and a T-cell activation domain were incorporated into the construct. Both of these generations expressed a single chain variable fragment (scFv) against CD19, which is expressed at a high-level on B cell malignancies. However, the second generation of CARs was more efficacious in showing antitumor effects in patients. The later generation of CAR T cells incorporated two co-stimulatory domains derived from different co-stimulatory domains [2], for purposes of enhancing the activation and proliferation of these cells upon interaction with their target antigen [3]. There have been more than 370 clinical trials on CAR T cells to date worldwide (clinicaltrials.gov, accessed on 29 June 2020) [4] and, although there are risks associated with CAR T cells, such as neurotoxicity and cytokine release syndrome (CRS), CAR T cells are the first case of cellular gene therapy to be commercially approved by the U.S. FDA. Figure 1 shows the general methodology of generating CAR expressing cells. Specifically, as the first step, leukocytes are extracted from the patient’s blood or donor’s body, and T cells are purified. Next, these T cells are genetically modified to express CAR using lentivirus (Kymriah^TM^) or retrovirus (Yescarta^TM^). After T cells are differentiated into their CD4 or CD8 T cells subsets, activation of the T cells is needed. CD8^+^ T cells can be activated with cytokines, such as IL-2 [5]. Interestingly, in addition to activation and proliferation of CAR CD8^+^ T cells, IL-2 has also been used clinically as a monotherapy to induce cancer regression in patients [6]. Importantly, the CARs are encoded with viral vectors, which integrate into the genome of the patient T cells, thus allowing them to bind directly to TAA, such as CD19, independent of HLA. However, the efficacy of CAR-T cell therapy is challenged by the nutrient depleted and immunosuppressive TME ensued by tumor cells. The high metabolic demand required for tumor cell proliferation and metastasis, as well as the increased ability for tumor cells to internalize nutrients, leaves the TME nutrient depleted [7,8]. Thereby starving effector T cells and preventing their anti-tumor cytotoxic effects [8]. This increased uptake of nutrients by tumor cells can lead to an increased accumulation of metabolic by-products, such as lactic acid and CO_2_, in the TME which in turn has been found to prevent effector T cell activity [9,10]. Additionally, tumor cells can evade the immune system via immunosuppressive mediators, such as immunosuppressive enzyme (Indoleamine-2,3-dioxygenase (IDO) or arginase) [6,11] or cytokine (Interleukin 10 (IL-10) or Transforming growth factor beta (TGFβ) [12,13] production for the induction of T cell suppression or tolerance. This here demonstrates the need to engineer highly resilient and metabolically fit CAR-T cells with capabilities of enduring the nutrient depleted and immunosuppressive TME.

In addition to CAR-T cells, bioengineering technologies have enabled great progress in developing other immune cell types such as CAR-NK cells, CAR-macrophages (CAR-M), and CAR-γδ T cells, which can provide effective responses in persistent solid tumors [14,15]. The following sections will discuss several bioengineering strategies that have led to the development of effective CAR T cell therapies, as well as the metabolic demand of anti-tumor CAR immune cells and an introduction to different types of non-T cell-based CAR therapies.

## 2. Challenges of CAR T Cell Therapy

Although, CAR T cells provide tremendous advantages in killing cancer cells, they also have drawbacks and mechanisms of resistance related to off-targeting effects and antigen loss of cancer cells. Antigen loss in certain cancers is likely contributed to antigen escape or lineage switch [16]. Antigen escape, which is occurs when there is a loss or downregulation of the target antigen, may take place when a patient relapses with a phenotypically similar cancer but lacks the expression of the previously targeted antigen [17]. For example, CAR T therapy can successfully kill one type of cancerous cell, but the patient may relapse if the tumor reforms with a different population of cancerous cells [18,19]. Monitoring CAR T cell efficacy for antigen loss may be essential for relapse prediction and prevention. In contrast, lineage switch can occur when a patient develops a genetically similar tumor with differences in phenotypic expressions [17].

In addition to antigen escape and lineage switch, unforeseen toxicities are another common limitation associated with CAR T cell therapies. The toxicity associated with T cell therapies may be related to incorrect dosages, off targeting effects, and incorrect timing of T cell activity. Specifically, CAR T cells can target healthy B cells in non-tumor tissues, and this can lead to “on-target, off-tumor” toxic responses. Additional mechanisms of resistance to CAR T cell therapy is the inability to harvest enough autologous T cells for CAR engineering, the inability to generate effective CAR technologies from patients who have previously been exposed to chemotherapy, and the inherent tumor heterogeneity being an obstacle in recognizing the most optimal target [20,21,22] Therefore, in order to overcome the drawbacks of CAR T cell therapies, further research needs to be done to identify multiple tumor-specific antigens, signaling domains, and optimizing and development of safe, reliable CARs, based on the specific type of tumor.

Notably, some of these challenges have been addressed pre-clinically using recent strategies of suicide genes, inhibitory CAR, dual-antigen receptors, or the use of exogenous molecules to help control CAR T cell function [23]. The implementation of these strategies have led to the development of more effective CAR T cell therapies [23]. Despite the deficiencies associated with CAR T cells, studies have clearly shown that CAR-based T-cell therapies can control tumor progression in patients that do not respond to conventional treatments [24,25].

Furthermore, three additional important parameters that should be considered when engineering CAR in T cells include (i) identifying the most relevant T cell subset to induce the most robust antitumor response, (ii) finding the best ex vivo T cell processing procedure to ensure that the most fit T cells are generated, and (iii) determining whether or not additional T cell engineering is required for the most optimal in vivo performance [26]. Each of these aspects require additional study for the further development of effective CAR cells that have a higher capability in targeting and killing cancerous cells within heterogeneous tumor complexes. Moreover, additional research on the manufacturing process of CAR cells can also decrease costs and increase the number of centers that specialize in engineering CAR constructs.

## 3. CAR T Cell Immunotherapy for Solid Tumors

Despite extensive efforts in pre-clinical studies, CAR T cell therapy has not been successful in treating solid tumors in clinic. There are several limitations to current CAR T cell technologies that need to be addressed in order to have a more efficacious construct when treating solid tumors. Namely, one of the limitations being the physical nature of solid tumors itself. The solid feature of the tumor creates a physical barrier, in turn preventing CAR T cells from successfully infiltrating the tumor. Consequently, this affects the CAR T cells’ ability to locate the ideal target antigen as compared to hematological diffused tumors [27]. Moreover, as observed in human tumor cultures, in order to access tumor sites and exert antitumor effects, CAR T cells must be able to degrade heparan sulphate proteoglycans (HSPGs) by releasing specific enzymes, such as heparanase (HPSE) in the TME to reach their target [28,29]. Notably, in solid tumors, chemokine-receptor mismatch, cancer associated fibroblasts (CAFs), physical barriers represented by the extracellular matrix (ECM) and stroma, and abnormal vasculature at tumor sites are also some of the limitations to CAR T cell infiltration [27]. In contrast to hematological cancers, where CAR T cells can circulate the bloodstream to eventually reach the targeted cancer cells without having to overcome physical barriers. Additionally, solid tumors promote infiltrating myeloid cells to produce immunosuppressive signals and molecules within the TME for the inhibition of T effector cell infiltration and activity [30]. Interestingly, a strategy of photothermal therapy has been shown to promote direct tumor cell killing, partial disruption of the ECM, and enhanced tumor infiltration and activation of CAR T cells in mice bearing human melanoma tumors [31]. Additionally, clinical studies have shown that CAR T cell infiltration within solid tumors can be enhanced when targeting a tissue-specific antigen, such as prostate-specific membrane antigen (PSMA), which can be found on malignant prostate cells [32]. However, the selective targeting of conventional CAR T cells is reliant on identifying specific TAAs of interest. Therefore, universal CAR-T cells have become a popular area of study and can promote the selective targeting of various antigens without prior TAA identification. For instance, given that CD16-CAR T cells are capable of identifying the FC-region of monoclonal antibodies, the combinatorial delivery of CD16-CAR T cells and monoclonal antibodies can promote the selective targeting of multiple antigens, and in turn avert the antigen loss, downregulation or mutation limitation that is associated with conventional CAR cell therapy [33,34,35,36].

In addition to trafficking and infiltration, multiple challenges in the hostile solid TME can affect the efficacy and function of CAR T cells. For example, nutritional depletion, acidic pH, oxidative stress, and hypoxia that are rendered by the metabolism of tumor cells, can also inhibit CAR T cell function [32,37]. Something that is also important to note when generating effective CAR T cells is to consider the reduction in memory and effector T cell activity in the TME due to (1) the clonal deletion of self/tumor-specific T cells leads to a decreased number in tumor-specific TCRs, (2) poor activation of innate immune cells and antigen-presenting cells (APCs) in the TME, and (3) formation of an overall immunosuppressive TME [38]. Interestingly, these challenges have inspired the development in CAR T cell-based treatments that partially overcome each of these three obstacles. However, the efficacy of CAR T cell therapy is influenced by multiple challenges generated by stromal cells, such as cancer associated fibroblast, and suppressive immune cells, tumor associated macrophages, tumor associated neutrophils, and Tregs. Other factors, such as immunosuppressive cells, the presence of inhibitory soluble factors, and cytokines are also responsible for hindering the ability of CAR T cells to target the solid tumors effectively.

The following sections discuss a few of these issues that are involved in reducing the efficiency of CAR T cells within solid tumors and the TME, as well as how metabolism plays a role in CAR T cell efficacy.

### 3.1. Impact of TME on T Cell Metabolism

Over the past few decades, the role of immune cell metabolism is being recognized as a major hurdle in limiting the function and efficacy of antitumor T cells for cancer therapy. The metabolic pathways within immune cells, in particular T cells, is known to control T cell activation, proliferation, differentiation, migration and function [39]. Therefore, recent efforts identifying that metabolites within the TME can alter T cell function is vital information for the future development of more stable and effective CAR technologies [40]. Additionally, hypoxia associated with the TME in solid tumors is one of the challenges that has been shown to decrease T-bet expression in TILs and reduce lymphocyte’s activation [41], and generation of high level of reactive oxygen species (ROS) by tumor cells can cause oxidative stress in mouse melanoma models. Therefore, such a TME can inhibit T cell immune responses, such as activation, proliferation, differentiation and apoptosis [42]. Interestingly, engineered CAR T cells have been generated to secrete an antioxidant enzyme, catalase (CAT), to reduce hydrogen peroxide to water and oxygen. Thus, these CAT-CAR T cells can maintain their anti-tumor function and were shown to have a reduced oxidative state with decreased levels in ROS accumulation in solid human tumor samples [32]. Moreover, since the metabolism of memory T cells relies on oxygen, hypoxic conditions are a major challenge for these cell types in the TME. Additionally, low oxygen concentrations can limit oxidative phosphorylation [27]. Studies have shown that increased levels of hypoxia can lead to an upregulation of PD-L1 and HIF-1a in myeloid-derived suppressor cells (MDSCs) to ultimately lead to T cell exhaustion and Treg generation [43].

Therefore, understanding the metabolic transition of T cells in the TME, and a change in the cellular metabolic reprogramming of cancer cells due to oncogenic mutations can lead to a better understanding of the issues related to the metabolic state in TME [44]. Glycolysis plays a crucial role in the differentiation and expansion of effector T cells. Upon encountering an antigen (such as lymphoma specific CD20) T cells undergo changes in their metabolic activity for their differentiation into effector cell subsets. Indeed, naïve T cells rely on oxidative phosphorylation (OXPHOS) and fatty acid oxidation (FAO) to meet energy demands. However, activated effector T cells rely on aerobic glycolysis to facilitate faster proliferation [44,45,46]. On the other hand, glycolysis is also a preferred metabolic program of cancer cells. An increased reliance on glycolysis over OXPHOS, known as Warburg effect, generates energy in the form of adenosine triphosphate (ATP) and lactate [44], under hypoxic conditions during the early avascular phase of tumor development [47]. Therefore, glucose availability in the TME is decreased due to the increased uptake by tumor cells, in turn, leading to lowered AKT and mTOR signaling in T cells, which are vital for a greater reliance on anabolic metabolism of T cells and their function. This then leads to a downregulation of glucose transporter (Glut1) and prevention of effector T cell activation and function [27,46]. Consequently, this process further diminishes an effector T cells ability to have an increased reliance on glycolytic metabolism. More recently, it has been shown that a reduction in glucose availability leads to a decrease of phosphoenolpyruvate, a glycolysis metabolite, which is necessary to sustain TCR signaling and antitumor T-cell effector activity [48]. Glucose depravation can also lead to an increased expression of programmed cell death protein 1 (PD-1) on T cells [49], however the inhibition of programmed cell death ligand 1 (PD-L1) on solid tumor cells, to prevent tumor-mediated T cell death, can drive tumor cells to rely more on OXPHOS. This in vivo data, within a sarcoma mouse model, suggested that this increased tumor reliance on OXPHOS may lead to an increase in glucose availability in the TME for effector T cell function and survival [49,50].

Interestingly, lactate, as a major byproduct of aerobic glycolysis is generated in large amounts in the TME and can hinder cytotoxic T lymphocyte activity and disturb T-cell metabolism [30,45,51]. Increased extracellular levels of lactate has shown to decrease the intracellular pH of T cells and inhibit T cell glycolysis, via direct inhibition of hexokinase 2 (HK) and 6-phosphofructo-2-kinase (PFK) [52,53]. Blocking acidification prior to anti-PD-1 or anti-CTLA-4 may lead to efficient anti-tumor responses [52]. Generation of lactate, and factors like vascular endothelial growth factor (VEGF), IDO, Prostaglandin E_2_ (PGE2), and adenosine are active players that contribute to the suppression of T cell immune responses within the TME. Moreover, low level of amino acids such as cysteine, arginine, tryptophan, and lysine within the TME can cause malfunctions in protein translation or can induce autophagy responses in effector T cells as well [37,54]. Low levels of arginine can alter T cell responsiveness due to the decreased expression of the CD3ζ chain. However, providing T cells with arginine has demonstrated an increase in pro-inflammatory cytokine production and an increase antitumor T cell responses in vitro [55,56]. Therefore, supplementing CAR-T cells with amino acids, such as cysteine or arginine, may lead to an increase in antitumor CAR T cell activity.

Notably, metabolic adaptation of cancer cells extends beyond ATP production. For example, cellular metabolism of several tumors can be modified by loss of tumor suppressors, such as P53, or activation of oncoproteins, such as phosphoinositide 3-kinase (PI3K). In fact, balance between energy production and macromolecular biosynthesis and redox status are key requirements of metabolic adaptation of tumor cells [44,47]. These factors lead to the immunosuppressive TME and low immunogenicity of cancer cells, which are ultimately responsible for restricting the therapeutic efficacy of CAR T cells in solid tumors. Thus, TME metabolism and immunometabolism is an active area of research to substantially improve clinical outcomes of CAR T immunotherapy for treating solid tumors. For example, to improve cell-based cancer immunotherapy, research has been performed on immune cell metabolism (e.g., T cells, dendritic cells, [57] macrophages) to understand how it is affected by the TME, and how it can be manipulated specifically in adoptive transfer therapies like CAR T immunotherapy [58,59,60,61].

**Figure 2:**
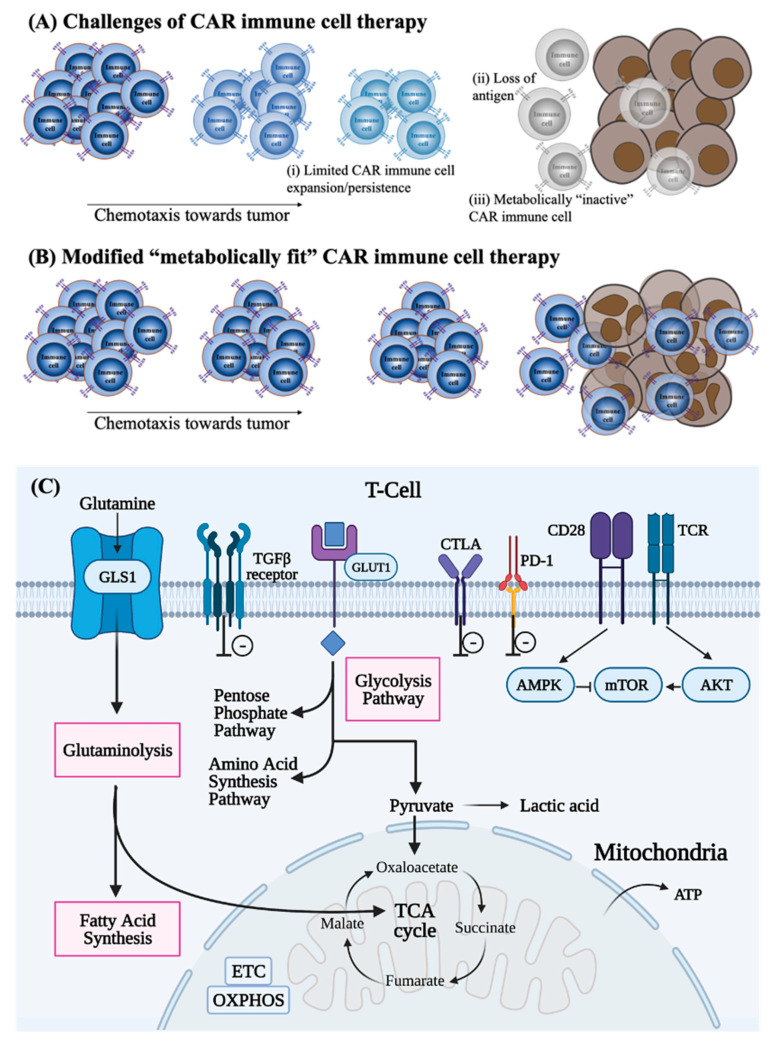
Metabolically fit chimeric antigen receptor (CAR) cells need to be generated for effective CAR immunotherapy. (**A**) When CAR immune cells reach their target, due to the paucity of nutrients, these cells can become exhausted. This prevents the CAR immune cells from functioning and allows for tumor growth. (**B**) Metabolically fit CAR immune cells can be generated by modifying the metabolic pathways that endow these immune cells to out-compete cancer cells for nutrients and thus remain active even in the TME for the eradication of cancer cells. (**C**) Metabolic pathways that are disrupted and can be modified to generate metabolically fit CAR-immune cells.

Interestingly, studies suggest that cancer cells outcompete T cells for glucose in vivo in cancerous mouse models, therefore preventing the cytokine production that is required for T cells to mount a cellular response against the tumor (Figure 2A) [50,62]. Although further studies are required to understand if this phenomenon is also consistent in human studies. However, it is observed that checkpoint inhibitor therapies (e.g., anti-CTLA-4) combined with other therapies are effective, and it is known that these checkpoint inhibitors accelerate glycolysis in TILs [63,64]. Therefore, this suggests that the ineffectiveness of antitumor T cells may be due to them being deprived of glucose in the TME. Notably, metabolic pathways diverge and converge at many different levels, and therefore, cells have to choose the most optimal path to achieve their metabolic goals to further determine their fate and function [65]. Overall, different metabolic pathway choices affect the function and fate of immune cells. Thus, metabolic commitment to a pathway is influenced by both signaling pathways and substrate availability in the microenvironment. These concepts have been applied to CAR T cell therapies for making these cells more effective in killing cancer cells in the solid TME (Figure 2B). For instance, inhibition of IDO because of increased tryptophan has shown promise for greater success in cancer treatment [66]. Similarly, checkpoint blockade therapy (anti-PD-1, anti-PD-L1, anti-CTLA-4) corrects nutrient restriction experienced by T cells in a progressing tumor by upregulating CD28 mediated glycolysis (Figure 2C) [50]. These elegant studies clearly demonstrate that metabolic regulation affects both the function of T cells and their response to low nutrient microenvironments [67]. These data also suggest that T cell function and cellular metabolism can be modified to treat different types of tumors in vivo [68]. Table 1 demonstrates how the TME modulates immunometabolism and potential strategies to overcome the induced metabolic impairments.

Nonetheless, the strategic selection of which co-stimulatory molecule is expressed by a CAR T construct can influence CAR T cell function and fate within the challenging TME. Several examples of T cell co-stimulatory molecules are CD28, ICOS (inducible T cell co-stimulator (CD278)), 4-1BB (CD137), OX40 (CD134), and CD27. The expression of a 4-1BB domain by a CAR T construct has previously demonstrated the induction of CD8+ central memory T cells with increased respiratory capacity and heightened mitochondrial biogenesis. In contrast, the incorporation of a CD28 domain has shown to stimulate effector memory T cell phenotypes with a gene signature signifying glycolytic metabolism [71,72]. Notably, a sustained activation, proliferation, and effector function in resting T cells, via the activation of NFκB, has been observed when combining a CD28 and OX40 domain to a CD3 ζ chain (CD28-OX40-CD3ζ) [73]. Nonetheless, ICOS can activate the PI3K-AKT signaling more effectively than CD28, which may elucidate a mechanism behind the increased T cell persistence within ICOS-based CAR T cells [74]. Furthermore, the incorporation of the CD27 domain, as a CAR co-stimulatory molecule, has exhibited a decrease in apoptotic pathways with an upregulation of B-cell lymphoma-extra large (BCL-(X) L), which is known to modulate metabolic functions of mitochondrial multiprotein complexes [71,75]. Therefore, the incorporation or combination of various intracellular signaling domains can alter the outcome of the CAR construct within the TME.

Among different approaches, one of the most promising approaches to modifying the TME in solid and hematological malignancies may be the combined delivery of CAR T therapies with existing protein therapies for an improvement of CAR T cell function. An example of such as approach is the constitutive expression of IL-12 by CAR T cells for an increased ability to eliminate cancer cells more effectively, which in turn leads to overcoming the immunosuppressive TME [76]. Therefore, generation modified CAR T cell immunotherapy, based on combinatorial engineering and treatments to reprogram T cell properties and the TME, can be unprecedented hope of therapeutic interventions for solid and hematological tumors.

### 3.2. Metabolic Engineering of CAR Cells

Metabolic engineering of CAR cells has a great potential to develop highly potent CAR T cells. For example, gene engineering approaches, such as overexpression of intercellular metabolic enzymes, [30] can improve CAR T cell activity in solid tumors. Overexpression of PPAR-gamma coactivator 1-α (PGC1-α), which programs mitochondrial biogenesis is one approach to potentially engineering the metabolism of CAR T cells. A defect in PPAR-gamma coactivator 1-α, due to chronic protein kinase B (Akt) signaling inhibiting Foxo transcription factor activity and consequent PGC1-α repression, can lead to loss of mitochondrial function in tumor-reactive T cells in the TME. Therefore, PGC1-α overexpressing T cells significantly increases mitochondrial mass, resulting in greater metabolic efficiency of T cells in the TME [77]. However, studies have not yet implemented this approach in CAR T cell therapies, indicating that further studies are required. Interestingly, another study showed that co-inhibitory factor gene editing, such as the combination of PD-1 blockade in CAR T cells [78] can also enhance CAR T cell function. Specifically, cancer cells often upregulate ligands, such as PD-L1, that bind to inhibitory receptors on T cells and limit the capacity of CAR T cells to combat solid tumors. Using clustered regularly interspaced short palindromic repeats (CRISPR)/Cas9 system to knockout PD-1 has been shown to augment the function of CAR T cells in vitro and in vivo. Indeed, CRISPR/Cas9 system can disrupt PD-1 gene locus in human primary T cells, which leads to reduction of PD-1^HI^ population. Notably, this reduction does not have a significant effect on CAR T cell proliferation. Besides the boosting of CAR T cell cytokine production, a combination of CAR T cells with CRISPR/Cas9-mediated PD-1 genome can enhance the ability of CAR T cells to recognize antigens and target antigen-expressing tumors [78]. Furthermore, the inhibition of PD-1 in T cells, is shown to lead to metabolic changes where T cells transition from glycolysis toward the Krebs cycle with an increased rate of FAO. This alteration in T cell metabolism using PD-1 inhibitors demonstrated an increase in T cell survival, function, and terminal differentiation by relying on a fat-based metabolism and in turn mimicking functions similar to memory T cells. PD-1 ligation has also been shown to enhance the PPAR/PPARγ PGC1-α axis when administering bezafibrate (a pan-PPAR agonist) for the prevention of T cell death and for the initiation of a long-lived T cell phenotype under PD-1 blockade [79,80,81]. Therefore, utilizing PD-1 therapies in conjunction with CAR T cell therapies may be a feasible approach to altering the metabolic profile of T cells as a strategy to maintain CAR T cell function in a nutrient depleted environment.

Adoptive cell therapy, such as TIL therapy, has also shown success and is a current clinical approach to treating cancer. In comparison to CAR-T cell therapy where circulating T cells from the blood are extracted and engineered to bind to specific proteins expressed by cancer cells, TILs are found and extracted from the tumor. TILs that recognize the tumor cells are then expanded and infused back into the patient. Although TIL therapy does not require engineering of T cells, TIL therapy is a more invasive approach and requires identifying TIL-rich tumor samples which may not exist or may be challenging to acquire [82]. Additionally, TILs have dysregulated metabolism due to the nature of the TME, which has shown to increase exhaustion and deplete effector T cell function [83]. Therefore, acquiring metabolically stable T cells from the periphery for CAR therapy may be an advantage in comparison to expanding metabolically dysregulated TILs for the treatment of cancer.

## 4. Other Potent CAR Immune Cells

The idea of generating metabolically fit immune cells can also be extended to other CAR immune cells such as NK cells, macrophages, and dendritic cells [84,85,86,87,88]. In fact, recent research is beginning to explore specific metabolic enzymes in these immune cell types, which can be manipulated to make these cells more metabolically fit. The metabolic reprogramming of macrophages and dendritic cells [89] has led to the discovery of metabolic processes, such as glycolysis, the Krebs cycle, and fatty acid metabolism, having significant effects on their cellular function [90]. For example, macrophages undergo metabolic reprogramming in response to environmental cues, danger signals, and cytokines. Macrophage function is also affected by certain metabolites such as succinate and citrate [90]. Overall, the immune system can regulate metabolic pathways to change cell function and fate, thus modulating these pathways in immune cells could generate a metabolically fit CAR-based immunotherapy.

### 4.1. NK Cell CAR Therapies

**Figure 3:**
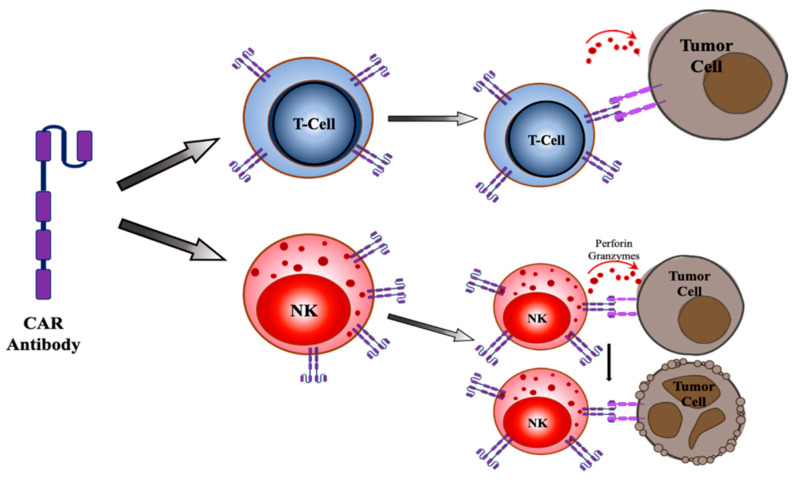
Targeting of tumor cells by T cells and NK cells. T cells and Natural Killer (NK) cells expressing a chimeric antigen receptor (CAR) recognize the antigens present on the tumor cells, bind them and release perforin and granzymes to directly initiate tumor cell death.

CAR NK cells have shown promising results for tumor suppression in pre-clinical testing. Successful pre-clinical tests of anti-CD19 CAR T cell therapy and remission of B cell malignancies has led to further investigations in CAR NK cells and its clinical applications (Figure 3) [87]. The inspiration for generating anti-CD19 CAR NK cells was to overcome the complexity in manufacturing CAR T cells and circumvent their associated toxicities [91]. About 73% of CD19-positive lymphoid tumor patients, who were treated with CAR NK therapy responded to treatment and approximately 88% of those patients had reached complete remission [91]. Additionally, patients had shown a response to the treatment within 30 days of the infusion, regardless of the dosage. Furthermore, CAR NK cells were active for at least 12 months in patients who received low doses [91]. CAR NK cells have also shown success in targeting solid tumors expressing antigens such as HER2, PSMA, mesothelin, ROBO1, or MUC1[92]. Importantly, a majority of the patients receiving CAR NK therapy had a positive response to the treatment, and CAR NK cells were not associated with any toxicity such as cytokine release syndrome, neurotoxicity, or graft-versus-host disease. Therefore, CAR NK cell immunotherapy presents an allogenic therapy that can be readily available for instant use [93,94]. Moreover, CAR NK cells are able to exert anti-tumor effects in addition to the CAR function since they also obtain their native receptors, therefore averting any relapse or resistance associated with antigen loss and CAR therapy [93,95,96]. Additionally, unlike CAR T cells, CAR NK cells can target tumor cells without the requirement of specific TAA recognition and despite the down-regulation of MHC class I on tumor cells [94,97]. This demonstrates the potential of CAR NK cells as universal CAR cells [93,98].

Although NK cells are effective phagocytic lymphocytes with high tumor suppressing activity, NK cells are functionally exhausted in the TME [99]. This is likely due to the nutrient and oxygen deprived TME that also consists of high levels of metabolic by-products, such as lactic acid. A reduction in IL-2-induced mitochondrial metabolism, such as OXPHOS and maximal respiration has been observed in human NK cells within the TME [100]. Nonetheless, the hypoxic environment can be used as an advantage when constructing NK cells. Juillerat et al. demonstrated that the incorporation of the oxygen-sensitive domain, HIF-1a, can generate a construct where the expression of CAR is reliant on low oxygen concentrations [101,102]. Additionally, CRISPR/Cas9 can be utilized to alter pathways that are involved in NK exhaustion or function [103]. For instance, CRISPR/Cas9 can successfully express the NKG2D ligand, major histocompatibility complex class I polypeptide-related sequence A (MICA), which may promote NK-mediated anti-tumor effects [104]. It is also important to note that tumor cells in the TME evade NK cell function via TGF-b, metabolic disturbances, and checkpoints among many other immunosuppressive mechanisms [96]. However, NK cells with a chimeric receptor consisting of the activating receptor, NKG2D, along with the cytotoxic ζ-chain of a TCR can overcome the immunosuppressive TME while promoting inflammatory responses within the TME [97,105]. Furthermore, transduction of NK cells with activating cytokines, such as IL-2, IL-12, IL-15, IL-18, and IL-21, can promote the proliferation and function of NK cells [106]. For example, NK cells co-expressing CAR and IL-15 were more potent than unmodified NK cells with and had shown to increase proliferative rates and selective cell-killing activity in breast carcinoma [107]. Furthermore, CAR NK persistence and function can be achieved when engineering memory-like NK cells with CAR [108].

Although CAR NKs have shown some success in recent clinical trials, there are still challenges associated with this treatment option. For example, CAR NK cells have low ex vivo expansion as well as low transduction efficiency and lifespan, which limits their use and warrants further research [94,109]. However, electroporation pulse codes and buffer optimization for protein uptake can improve NK transduction rates [103]. Nonetheless, CAR NK cells may have several advantages over CAR T cells including safer clinical uses, more advanced mechanisms of cancer cell recognition, and an increased abundance of NK cells in clinical samples for the generation of CAR NK cells [109].

### 4.2. CAR Macrophages

Due to the success of CAR T cell and CAR NK cell therapies in the clinic, further research has been carried out to engineer other potent CAR immune cells in pre-clinical animal models. Among those, a promising cell type, that is gaining traction in CAR-based immunotherapy, is CAR macrophages. Engineering CAR macrophages is a relatively new avenue for CAR research which attempts to overcome some of the previously mentioned limitations associated with CAR T therapies.

Macrophages are known to naturally traffic into solid tumors and may result in a targeted cancer cell treatment that leaves healthy cells unaffected. Interestingly, a family of engineered chimeric antigen receptors for phagocytosis (CAR-Ps) has recently been generated and might direct macrophages towards the desired cancerous cells targets [88]. CAR-P macrophages have shown specificity toward targets as they have been shown to recognize and attack beads coated with the CD19 protein [88]. Furthermore, CAR-P macrophages have been able to phagocytose cancer cells and debris in vitro as well.

In another interesting development, human epidermal growth factor receptor 2 (HER2) targeting-CAR-Ms were developed with the capacity to phagocytose HER2 antigen expressing ovarian cancer cells [14]. Moreover, it was found that a one-time combined treatment of CAR-Ms and T cells decreased tumor burden in a xenograft mouse models. Interestingly, the infusion of CAR-Ms in mice converted the M2 (immunosuppressive macrophage phenotype) type of macrophages in the tumor to M1 (pro-inflammatory macrophage phenotype) [14] and induced antigen specific T cell responses against the tumors. Interestingly, in 2018, Carisma Therapeutics was successful in raising funds for developing CAR macrophage immunotherapies. In addition to CAR-macrophages, the precursor of macrophages, monocytes can also have antitumor activity. The advantage of using monocytes as oppose to macrophages can be that it reduces the time between retrieval and infusion from seven days to one day.

Overall, CAR monocytes/macrophages are a promising avenue of CAR cell-based immunotherapy and has the potential to overcome the shortcomings of CAR T cell-based immunotherapies, especially in targeting solid tumors.

## 5. Summary

In summary, cellular metabolism plays a crucial role in the immune response and based on different stages of immune cell phenotype (naive, effector, memory, regulatory T cells; M1; M2) and their activation state, the metabolic properties of these cells will change. Additionally, other factors, such as nutrients, cytokines, and growth factors, can affect effector T cell metabolism. Moreover, the high glycolytic metabolism of tumor cells creates a microenvironment that is low in vital nutrients, in turn making it highly hypoxic and acidic, which then leads to the metabolic inhibition of immune cells, poor inflammatory cell trafficking to the tumor, the production of immunosuppressive cytokines, and expression of co-inhibitory ligands. These suppressive influences render significant challenges for CAR cell therapies. Moreover, although several strategies have been tested to tackle solid tumor barriers, such as the use of alternative cytoplasmic activation domains and the use of CRISPR-Cas9 as gene-editing techniques, these need to be validated in clinic. Notably, combination therapy with checkpoint inhibitors and armed CARs has been used to improve the function of CAR T cells in solid tumors and are being tested in clinical trials. Importantly, new strategies are required to improve the metabolic fitness of CAR T cells within the TME and strategies are also required to improve the safety of CAR cells, particularly as they move into clinic. Indeed, CAR cells can be optimally designed based on the metabolic properties of the tumor being targeted, and cultured to promote a less differentiated, long-lived phenotype that can efficiently self-renew and differentiate in vivo into potent effector cells. Next-generation CAR cell immunotherapy based on combinatorial engineering and treatments to reprogram immune cell properties and the TME offer unprecedented hope of therapeutic interventions for solid tumors.

## 6. Future Directions

Generating highly pure and metabolically fit CAR immune cells is a major challenge, and precision genetic modulation of metabolic pathways may improve efficacy toward treating solid tumors. Moreover, pharmaceutical modification of CAR immune cells can also be utilized to modify these energy metabolic pathways to drive the activation of CAR immune cells, specifically for the treatment of solid tumors. These strategies may pave the way to more efficient CAR therapies against solid tumors.

A major issue that needs to be addressed for CAR therapy is the costs associated with manufacturing. Engineering strategies of non-viral vectors, developing protocols for in-hospital CAR therapy generation and generating CAR expression in non-T cells are some of the approaches that may lead to a decrease in these costs. Furthermore, a significant investment in engineering principles and omics approaches are needed to improve cellular manufacturing and the quality control and assurance of CAR therapies. The next stages in developing CAR immune cells will require marrying the fields of engineering and gene therapy for increasing the efficacy of treatment of solid tumors with low toxicity.

## Figures and Tables

**Table 1:** Summary of TME mediated modulation of immunometabolism.

TME Factors Affecting Immune Cell Metabolism	Direct and Indirect Metabolic Impairment of Immune Cells in TME	Strategy to Overcome Induced Metabolic Impairment	References
Increased hypoxia	Increases HIF1-a and PD-L1 on MDSCs for T cell exhaustion and Treg generation	Anti-PD-L1 and HIF inhibitors	[43,69,70]
Increased reactive oxygen species	Oxidative stress-mediated inhibition of NF-kB or mTOR for the prevention of T cell activation	Catalase, an antioxidant enzyme	[32,42]
Decreased glucose availability	Reduced AKT, mTOR, GLUT1, phosphoenolpyruvate, and increased PD-1 expression in T cells	anti-PD-1, anti-PD-L1, anti-CTLA-4	[27,46,48,49,50]
Increased lactate	Inhibition of T cell glycolysis and function	Blocking acidification prior to anti-PD-1 or anti-CTLA-4 administration	[52]
Low levels of arginine	Reduced responsiveness of T cells due to decreased expression of CD3ζ chain	Administering arginine	[55,56]

## Data Availability

Not applicable.

## Abbreviations:

ACT	Adoptive cell transfer
AKT	protein kinase B
APCs	Antigen presenting cells
ATP	Adenosine triphosphate
B-ALL	B cell acute lymphoblastic leukemia
Bcl-X(L)	B-cell lymphoma-extra large
CAFs	Cancer-associated fibroblasts
CAR	Chimeric antigen receptor
CAR-M	Chimeric antigen receptor macrophage
CAR-Ps	Chimeric antigen receptors for phagocytosis
CAT	Catalase
CCR	Chimeric costimulatory receptor
CLL	Chronic lymphocytic leukemia
CRISPR	Clustered Regularly Interspaced Short Palindromic Repeats
CRS	Cytokine release syndrome
ECM	Extracellular matrix
ETBR	Endothelin B receptor
FAO	Fatty acid oxidation
GLUT1	Glucose transporter 1
HER2	Human epidermal growth factor receptor 2
HK	Hexokinase 2
HPSE	Heparinase
HSPG	Heparan sulphate proteoglycans
ICOS	Inducible T cell co-stimulator
IDO	Indoleamine-2,3-dioxygenase
IL-10	Interleukin 10
LBCL	Large B cell lymphoma
MDSC	Myeloid-derived suppressor cells
MICA	Major histocompatibility complex class I polypeptide-related sequence A
NK	Natural killer
OXPHOS	Oxidative phosphorylation
PD-1	Programmed cell death protein 1
PD-L1	Programmed cell death ligand 1
PFK-6	phosphofructo-2-kinase
PGC1-α	PPAR-gamma coactivator 1-α
PGE2	Prostaglandin E2
PI3K	Phosphoinositide 3-kinase
PSMA	Prostate-specific membrane
ROS	Reactive oxygen species
ScFv	Single chain variable fragment
TAA	Tumor-associated antigens
TCR	T cell receptor
TGFβ	Transforming growth factor beta
TILs	Tumor-infiltration lymphocytes
TME	Tumor microenvironment
VEGF	Vascular endothelial growth factor
